# Reduced bone length, growth plate thickness, bone content, and IGF-I as a model for poor growth in the CFTR-deficient rat

**DOI:** 10.1371/journal.pone.0188497

**Published:** 2017-11-30

**Authors:** Michael S. Stalvey, Viktoria Havasi, Katherine L. Tuggle, Dezhi Wang, Susan Birket, Steve M. Rowe, Eric J. Sorscher

**Affiliations:** 1 Department of Pediatrics, University of Alabama at Birmingham, Birmingham, AL, United States of America; 2 Gregory Fleming James Cystic Fibrosis Research Center, University of Alabama at Birmingham, Birmingham, AL, United States of America; 3 Cystic Fibrosis Foundation, Bethesda, MD, United States of America; 4 Department of Pathology, University of Alabama at Birmingham, Birmingham, AL, United States of America; 5 Department of Medicine, University of Alabama at Birmingham, Birmingham, AL, United States of America; 6 Department of Pediatrics, Emory University, Atlanta, GA, United States of America; INSERM, FRANCE

## Abstract

**Background:**

Reduced growth and osteopenia are common in individuals with cystic fibrosis (CF). Additionally, improved weight and height are associated with better lung function and overall health in the disease. Mechanisms for this reduction in growth are not understood. We utilized a new CFTR knockout rat to evaluate growth in young CF animals, via femur length, microarchitecture of bone and growth plate, as well as serum IGF-I concentrations.

**Methods:**

Femur length was measured in wild-type (WT) and SD-*CFTR*^*tm1sage*^ (*Cftr-/-*) rats, as a surrogate marker for growth. Quantitative bone parameters in *Cftr-/-* and WT rats were measured by micro computed tomography (micro-CT). Bone histomorphometry and cartilaginous growth plates were analyzed. Serum IGF-I concentrations were also compared.

**Results:**

Femur length was reduced in both *Cftr-/-* male and female rats compared to WT. Multiple parameters of bone microarchitecture (of both trabecular and cortical bone) were adversely affected in *Cftr-/-* rats. There was a reduction in overall growth plate thichkness in both male and female *Cftr-/-* rats, as well as hypertrophic zone thickness and mean hypertrophic cell volume in male rats, indicating abnormal growth characteristics at the plate. Serum IGF-I concentrations were severely reduced in *Cftr-/-* rats compared to WT littermates.

**Conclusions:**

Despite absence of overt lung or pancreatic disease, reduced growth and bone content were readily detected in young *Cftr-/-* rats. Reduced size of the growth plate and decreased IGF-I concentrations suggest the mechanistic basis for this phenotype. These findings appear to be intrinsic to the CFTR deficient state and independent of significant clinical confounders, providing substantive evidence for the importance of CFTR on maintinaing normal bone growth.

## Introduction

Cystic fibrosis (CF) is a multi-organ disease of genetic origin. Among Caucasians, CF is the most common life-limiting recessive genetic disorder [1]. The disease is caused by mutations in the cystic fibrosis transmembrane conductance regulator (CFTR) gene, which produces a dysfunctional ion channel in CF patients and results in multiple sequelae. A major manifestation that characterizes individuals with CF, is the inability to absorb fats and other nutrients from the intestine. A second manifestation is poor lung function, attributed to chonic airway inflammation and infection. Over the past 40 years, life expectancy of children with CF in the United States has increased to over 40 years [1]. This change is attributed to improvements in airway clearance, antibiotics, and nutritional support. Nevertheless, despite these improvements, short stature remains a common occurence in CF and adversely affects total lung function.

It is not yet known whether problems with bone health in CF are interconnected to abnormalities of growth, and the CFTR dependence of these observations. Osteopenia and osteoporosis combined—referred to as CF-related bone disease or CFBD—have a prevalence of 17% of all patients in the CF Foundation Patient Registry, and is a major impediment to quality of life and continued health of CF adolescents and young adults [1]. Bone health involves growth and formation, in childhood and adolescence, evolving into a continued dynamic interaction of bone reabsorption and new bone formation in adulthood [2]. Evidence, in both animal and human studies, supports a defect in CFBD stemming from both excessive bone resorption and decreased bone formation [2].

Nevertheless, the study of growth restriction and bone health in CF is complicated by clinical factors that can also impact growth indirectly [2, 3]. Although nutrition is clearly a factor, clinical data indicates that there is a problem beyond this. A problem in early infant growth emerges, irrespective of weight. CF infants with pancreatic insufficiency demonstrate a reduction in mean z-scores for weight and length by 3 months of age. By 12 months of age, weight has been restored to healthy newborn, however length is not [4]. In general, CFBD in human subjects is characterized by diminished linear growth velocity, diminished final height (as well as weight), and decreased IGF-I [5–7]. Interestingly, abnormalities in growth and bone content—resembling the clinical scenario—have also been noted in the studies of animal CF models. CF mice and pigs have decreased growth early in life and reduced IGF-I concentrations [8, 9]. Additionally, CF mice have reduced bone content as young as three weeks of age, which continues well into adulthood [10, 11]. The similarities to the clinical phenotype make animal models an invaluable resource in the study of CFBD.

Recent reports suggest abnormalities of *in vitro* bone dynamics occur due to CFTR deficiency [12]. However, the abnormalities in osteoblast and osteoblast-osteoclast interactions do not explain reduced growth, and have not been well studied in previous animal models. We hypothesize that an inherent defect exists in growth and bone metabolism in the absence of normal CFTR function. To further understand the role of defective CFTR on bone growth and metabolism, we utilized the cystic fibrosis rat model. The CF rat allows for examination of bone health focused on CFTR-specific defects, rather than interference by other clinical confounders, such as pancreatic insufficiency. We discovered that CF rats, despite the absence of overt lung disease and adequate pancreatic function, exhbit a clear defect in bone growth localized to the growth plate that is attributed to IGF-1 deficiency. These results indicate abnormal growth is a CFTR-specific manifestation and potential therapeutic target in the disease.

## Methods

### Animals utilized

All experiments were performed at University of Alabama at Birmingham (UAB) under the auspices of the Institutional Animal Care and Use Committee (IACUC) approved protocol (IACUC-09479). The SD-*CFTR*^*tm1sage*^ (*Cftr-/-*) rat, developed through a collaboration between SAGE Labs and UAB using ZFN endonuclease gene editing technology (previously described in Tuggle et al.), was utilized for this study [13]. *Cftr-/-* rats were generated by microinjection of *Cftr* ZFN mRNA microinjection into fertilized eggs. Injected eggs were implanted into pseduopregnant female rats to establish the founder animals.

Male and female Sprague-Dawley (*Cftr+/-*) rats were paired and provided food and water ad libitum. Litters were genotyped during the first week of life by extracting tail snip genomic DNA, amplifying DNA using PCR (forward primer 5’-GCAGCTCACTGGTCGATCTT, reverse primer 5’-GACACTATATTCACAAGGGAGAG), and resolving PCR products on a 3.5% agarose gel to distinguish the 172-bp wild-type *Cftr* fragment from the 156-bp DNA fragment of mutant *Cftr*. Litters remained with lactating dams until weaning at 3 weeks of age. At weaning, wild-type (WT) and CFTR deficient (*Cftr-/-*) animals were provided DietGel 76A with casein (Clear H2O, Westbrook, ME) in addition to water and regular rodent chow (pellet and ground). To further reduce complications associated with intestinal obstruction in the animal model, GoLYTELY was added to drinking water (50:50 ratio) in at weaning. For this study, approximately half of the animals in each group received GoLYTELY supplementation. Details on the husbandry conditions and characterization of CF-related phenotypes are detailed in Tuggle et al [13].

### Necropsy procedures

Animals were euthanized via i.p. injection with sodium pentobarbital and blood collected for serum analysis. Both femora from each animal were stripped of musculature and placed in 10% phosphate-buffered formalin (pH 7.4) for 24 hours for tissue fixation. These bone specimens were then transferred to 70% ethanol and processed as described below.

### Ex vivo bone structural analyses

Femur length measurements were performed using a Fowler 0–6" range digital caliper (Cole-Parmer Instrument Company). Excised rat femurs were scanned using the Scanco mCT40 desktop cone-beam micro-CT scanner (Scanco Medical AG, Brüttisellen, Switzerland). The femur was placed inverted in a 16mm diameter scanning holder and scanned at the following settings: 16mm resolution, 70kVp, 114μA with an integration time of 200ms. Scans were automatically reconstructed into 2-D slices and all slices were analyzed using the mCT Evaluation Program (v5.0A, Scanco Medical). For the determination of cortical bone, 50 slices were scanned at the midshaft of the bone and the region of interest (ROI) was drawn on every slice and fitted to the outside of the cortical bone, to include all the bone and marrow. The threshold for cortical bone was set at 306. The 3-D reconstruction was performed using all the outlined slices. Data were obtained on bone volume (BV), total volume (TV), BV/TV, bone density and cortical thickness. For the determination of trabecular bone, the scan was started distal to the growth plate and consisted of 310 slices. The ROI was outlined starting below the growth plate and extending 200 slices (3.2mm), on the inside of the cortical bone, enclosing the trabecular bone and marrow. Trabecular bone was thesholded at 220 and the 3-D analysis performed on the 200 slices. Data were obtained on bone volume, density, total volume, connective density, trabecular number, thickness and separation.

### Quantitative bone histomorphometry

Rat femurs were fixed, processed, and infiltrated by methyl methacrylate (MMA) mixture prior to MMA embedding. Histologic processing was performed by increasing concentrations of alcohol, beginning at 70% and progressing to 100% over a total of 19 hours, followed by xylene. Post-processing was then performed with MMA. 5 um sections of MMA embedded mineralized bone from the distal femora were cut in the coronal plane using a Leica 2265 microtome. Sections were then stained with Goldner’s Trichrome. Standard bone histomorphometry was performed by methods of Parfitt et al [14] using Bioquant Image Analysis software (R & M Biometrics, Nashville, TN). All measurements were performed on one slide, by two independent evaluators in the histopathology core. The ROI for these samples was set at 250 microns off of the growth plate and endosteum with a depth of 2 millimeters. Using a two-dimensional histological section displaying profiles of three-dimensional structure, four types of primary measurements were made–area, length (or perimeter), distance between points or line, and number. Tissue volume, bone volume, bone surface, and osteoid surface were used to derive indices such as trabecular number and trabecular space. The cartilaginous growth plate was evaluated based on overall thickness, as well as direct measurements of the proliferative zone and hypertrophic zone [15]. Processing, embedding, and analysis of samples was performed by the Histomorphometry and Molecular Analysis Core in the UAB Department of Pathology.

### ELISA

IGF-I concentrations were determined in serum collected from additional rats (both *Cftr-/-* and WT) following euthanasia. The assay was conducted using the Mouse/Rat IGF-I Quantikine ELISA from R&D Systems. Sera were 1000x diluted for analyses. Samples were run in duplicate together with standard dilutions and control samples.

### Statistical analysis

Statistical analyses were conducted using GraphPad Prism 6.0 software (GraphPad Software, La Jolla, CA) and SAS version 9.4 (SAS Institute Inc., Cary, NC) Group differences in length were analyzed via general linear model, adjusted for age and gender. Data on bone content, histomorphometry and serum IGF-I concentrations were analyzed via an unpaired t test, and results reported as mean values and standard error, with a two-tailed p < 0.05 considered significant. To further evaluate the influence of bone size on the outcomes of bone content, a general linear model included length in addition to age and gender, and where applicable age by group interaction. Additionally, as a sensitivity analysis, models were run adding the use of GoLYTELY in the drinking water.

## Results

### *Cftr-/-* genotype results in decreased growth

**Fig 1:**
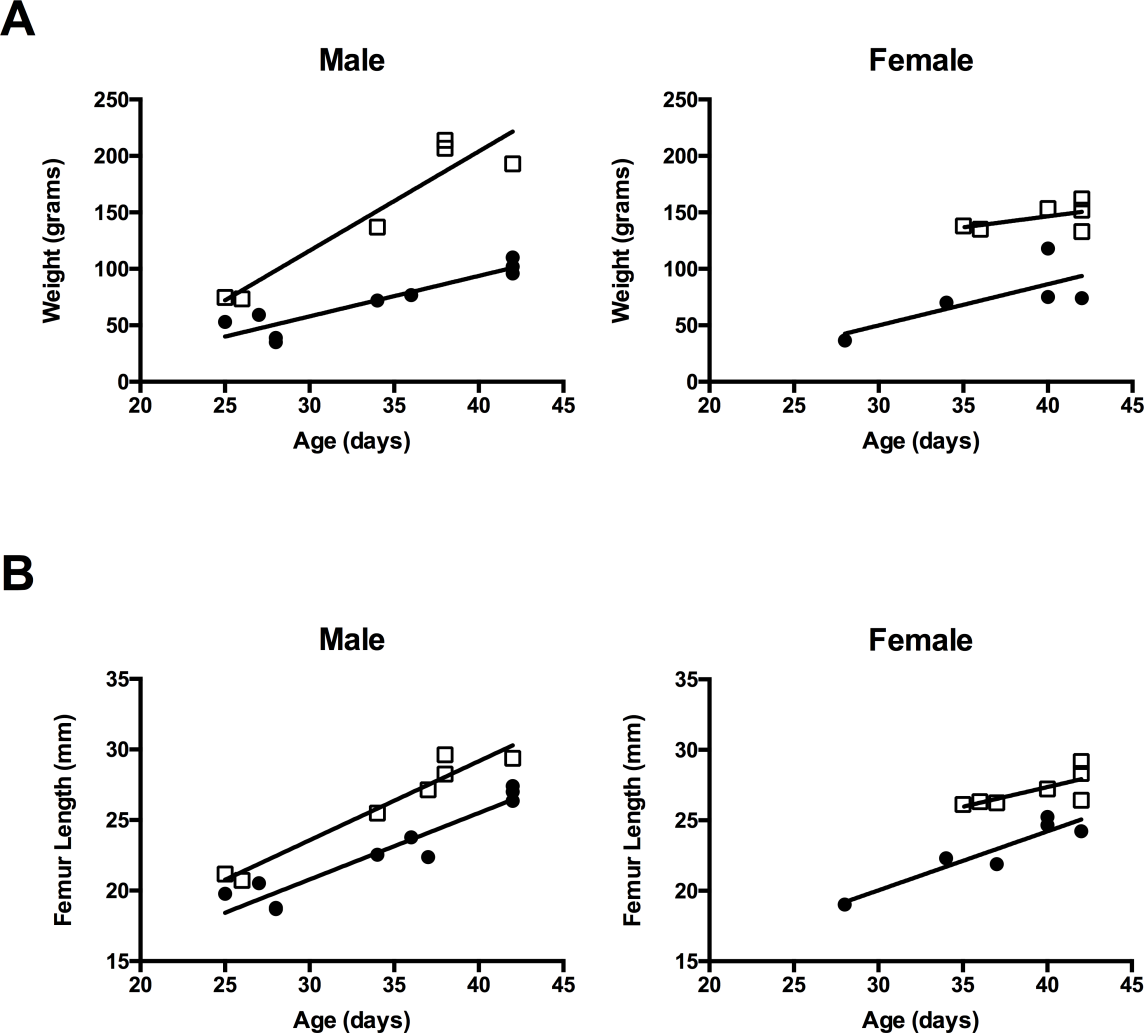
*Cftr*-/- rats have reduced size and femur length. **(**A) *Cftr-/-* rats are notably smaller in size compared to their wild type (WT) littermates. Animal total body weights are demonstrated versus age of the animals in *Cftr-/-* rats (•) compared to WT rats (▯). These findings are consistent with previously published data by Tuggle et al [13]. (B) Excised femurs obtained at sacrifice were measured by digital calipers. Femur length is reduced in *Cftr-/-* rats (•) compared to WT rats (▯) regardless of age or gender (p<0.0001).

In the 3–6 week old CF rats evaluated in this study, visible differences in overall size versus WT littermates were noted. Weight of *Cftr-/-* rats is similar to littermates at birth, however by two weeks of age, a separation in weight gain is noted between *Cftr-/-* and WT rats (Fig 1) [13]. We compared femur length of WT and *Cftr-/-* rats following measurement with precision calipers. To adjust for different ages and gender of the animals, measurements were analyzed utilizing a general linear model. Regardless of age or gender, femur length was consistently shorter in *Cftr-/-* rats compared to WT (p<0.0001)(see Fig 1). This difference persisted in the rats (p<0.0001), regardless of the absence or addition of GoLYTELY (Braintree Laboratories Inc., Braintree, MA) in the water supply to prevent intestinal obstruction.

### *Cftr-/-* rats have reduced bone content

**Fig 2:**
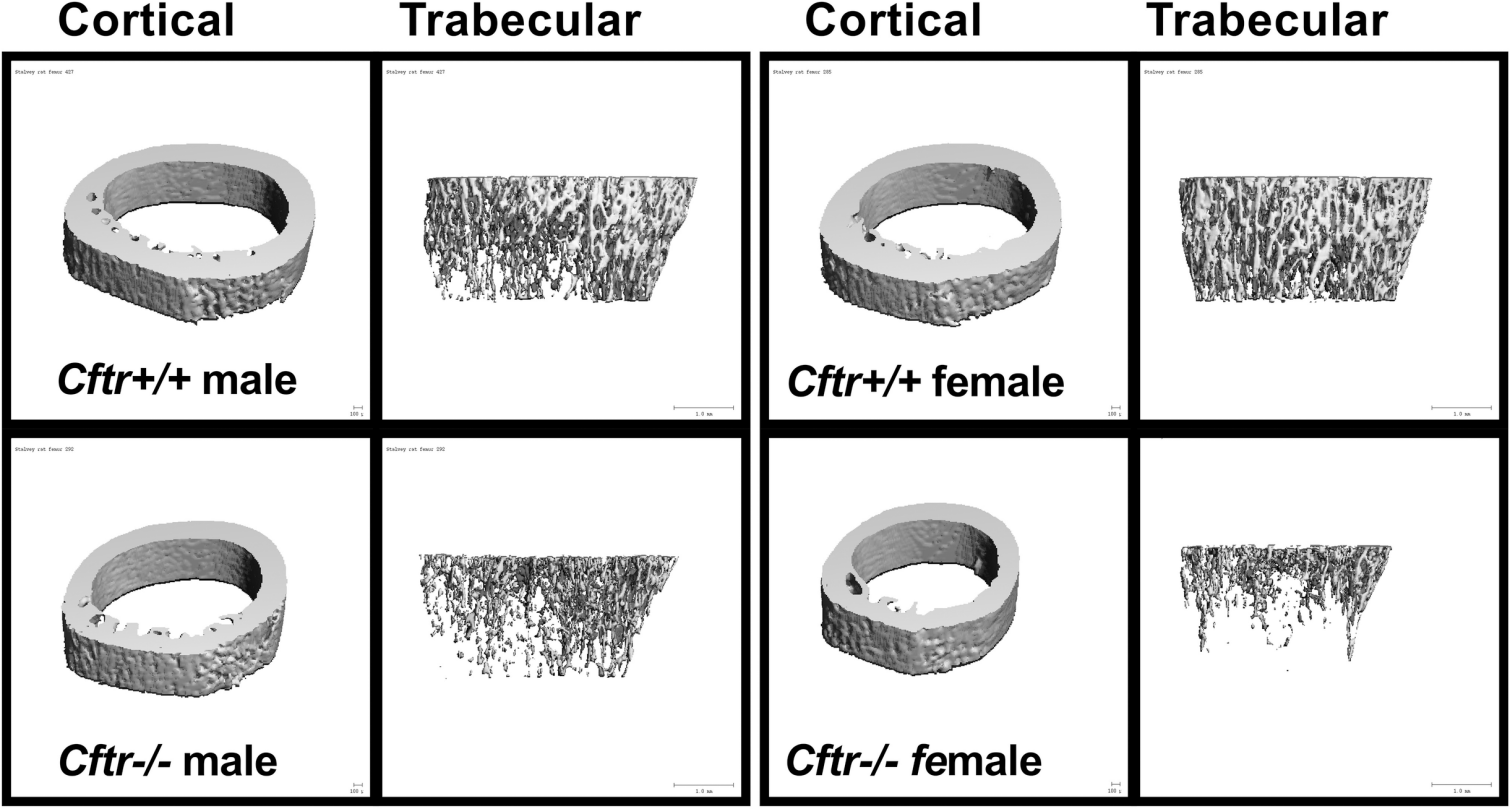
Micro CT imaging demonstrates reduced bone content. Representative 3D micro CT images of cortical and trabecular bone structures from young *Cftr-/-* and WT rats. MicroCT images demonstrate reduced bone content in *Cftr-/-* rats (more predominantly in females and detailed in Table 1). MicroCT images obtained from 38 day old WT and 42 day old *Cftr-/-* male rats. Female rat microCT images are from 42 day old *Cftr-/-* and WT rats. MicroCT images are from the male and female rat femurs pictured in histology images (Figs 3 and 4).

Bone volume for all groups was evaluated via micro-CT and reported for both trabecular and cortical bone segments. Comparisons between groups based on gender alone can be found in Table 1 and Fig 2. Trabecular total volume was reduced in *Cftr-/-* male and female rats compared to WT (reduced 19% and 29% respectively, p<0.05 and p<0.0001). In addition, cortical bone total volume was reduced in *Cftr-/-* male and female rats compared to WT (reduced 21% and 26% respectively, p<0.05 and p<0.0001). The reduction in measurements of bone content were greater in female *Cftr-/-* rats (see Table 1 and Fig 2). Female *Cftr-/-* rats demonstrated a reduced trabecular bone volume (p = 0.0001), trabecular bone volume/total volume (p = 0.0004), trabecular number (p = 0.0009), trabecular thickness (p = 0.0151) and increased trabecular spacing (p = 0.0017). Female *Cftr-/-* rats also had reduced cortical bone volume (p = 0.0015), however cortical thickness was increased (p = 0.0013), suggesting smaller overall bone. Connective density was also reduced in *Cftr-/-* rats regardless of animal sex (p = 0.0002 for females; p = 0.047 for males). Male *Cftr-/-* rats demonstrated reduced trabecular bone volume (p = 0.005) and trabecular bone volume/total volume (p = 0.02). However, there were no significant differences in trabecular number, thickness or spacing for male rats. Since trabecular bone is the most actively dynamic bone, and given the more pronounced reductions seen in female rats, less significant findings in male rats would imply that there is decreased formation or increased resorption of bone in female rats. Cortical thickness was also increased in male *Cftr-/-* rats (p = 0.03). Trabecular bone density and cortical bone density were not significantly different in either male or female rats. These findings indicate a reduced bone size and overall content of the bone, but not a reduction in bone density.

**Table 1:** Micro CT of femurs.

Measurement (n)	Female WT (7)	Female *Cftr-/-* (6)	Male WT (7)(7)	Male *Cftr-/-* (10)
Trabecular total volume (mm^3^)	19.37±0.8	13.81±0.5****	19.37±1.4	15.76±0.9*
Trabecular bone volume (mm^3^)	2.49±0.2	0.81±0.1***	1.46±0.3	0.66±0.1**
Trabecular bone volume/total volume	0.13±0.01	0.06±0.01***	0.073±0.01	0.042±0.01*
Trabecular number (1/mm)	2.85±0.2	1.54±0.1***	1.72±0.2	1.45±0.1
Trabecular thickness (mm)	0.057±0.001	0.051±0.002*	0.051±0.002	0.049±0.001
Trabecular spacing (mm)	0.38±0.04	0.70±0.07**	0.63±0.05	0.76±0.06
Trabecular bone density (mgHA/cm^3^)	899.5±7	881.5±19	866.8±12	855.0±20
Cortical total volume (mm^3^)	4.89±0.1	3.58±0.2****	4.80±0.3	3.82±0.3*
Cortical bone volume (mm^3^)	2.26±0.1	1.49±0.2**	1.92±0.3	1.31±0.2
Cortical bone volume/total volume	0.46±0.02	0.41±0.03	0.39±0.03	0.33±0.03
Cortical thickness (mm)	0.81±0.02	1.14±0.08**	0.81±0.05	1.07±0.08*
Cortical bone density (mgHA/cm^3^)	1231±1	1228±2	1186±2	1185±2
Connective density (1/mm^3^)	79.11±4.2	34.00±7.2***	38.83±5.7	22.83±4.7*

(*p <0.05, **p <0.01, ***p <0.001, ****p <0.0001)

To evaluate the impact of bone size on bone content, outcomes were analyzed by a general linear model, adjusting for length, age, gender and where applicable age by group interaction. Despite the adjustment for smaller sized bones, *Cftr-/-* rats still demonstrated reduced trabecular bone volume (p<0.0001) and trabecular bone volume/ total volume (p = 0.0017). Additionally, there was a reduction in connective density (p = 0.03) that persisted despite the length of the bone. As a sensitivity analysis, the model was re-analyzed with and without the addition of GoLYTELY in the drinking water to augment intestinal function by preventing obstruction. These differences in bone volume persisted (p<0.05 for trabecular bone volume, trabecular bone volume/total volume, and connective density) regardless of the presence or absence.

### Quantitative bone histomorphometry

**Fig 3:**
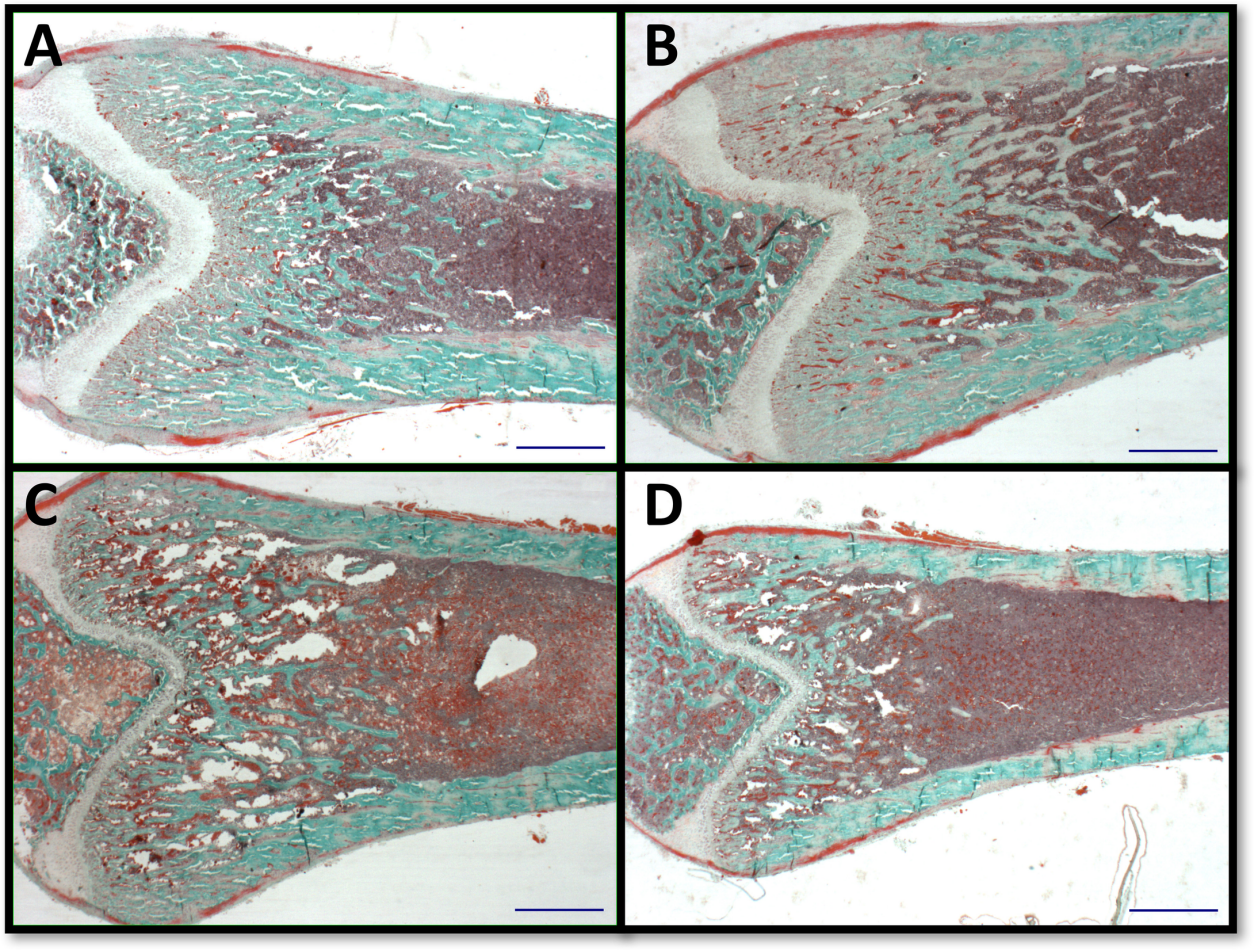
Histomorphometric analysis. Representative histology images demonstrate histomorphologic findings of reduced bone content in *Cftr-/-* rats (more predominantly in females and detailed in Table 2). Each figure demonstrates a blue line of 1000 microns in length for size consistency and photographed at 1.25X magnification. Pictured images obtained from 38 day old WT (A) and 42 day old *Cftr-/-* (C) male rats. Female bone images are from 42 day old *Cftr-/-* (B) and WT (D) rats. Histology images are from the male and female rat femurs pictured in microCT images (Fig 2).

Next we evaluated the histological content of femurs to define the cause of growth arrest. Consistent with micro CT evidence of reduced bone density, bone histomorphometry of the distal femur also revealed more prominent reductions in bone content in female *Cftr-/-* rats (Table 2 and Fig 3). Both bone area and total tissue area, as well as bone area/total area, were reduced in *Cftr-/-* females compared to WT female rats (p = 0.001, p = 0.03, and p = 0.0005). Trabecular thickness and number were reduced, and trabecular spacing was consequently increased, in *Cftr-/-* female rats (p = 0.0191, p<0.0001, and p = 0.0007). Bone perimeter was reduced in female *Cftr-/-* rats (p = 0.0006), as well as measurements of erosion perimeter, quiescent perimeter, osteoblast and osteoclast perimeters (p = 0.0024, 0.0007, 0.0005, and 0.0024). When compared against the bone perimeter, the reduction of osteoblast perimeter to bone perimeter in female *Cftr-/-* rats persisted (p = 0.0118). Findings in males were similar, although exhibited some important differences. Male *Cftr-/-* rats demonstrated a reduction in trabecular number (p = 0.0199). Although male rats demonstrated reduced erosion perimeter, osteoblast and osteoclast perimeters (p = 0.0294, 0.0345, and 0.0293), no difference in the ratio to total bone perimeter was observed. These findings indicate a reduction in size that is more apparent in female rats, as well as a reduction in the metabolically active bone. The authors acknowledge the small number of animals per group may lack the power to detect differences noted in the histological analysis. In an attempt to control for this, a second slice of each specimen was examined by a blinded reviewer. The ROI studied in the second section was within 5 microns of the detailed result, and was consistent with the above findings.

**Table 2:** Femur histomorphometry.

Measurement (n)	Female WT (7)	Female *Cftr-/-* (5)	Male WT (6)	Male *Cftr-/-* (7)
Bone Area (BV) mm^2^	1.83±0.21	0.58±0.13**	0.86±0.07	0.58±0.16
Tissue Area (TV) mm^2^	5.94±0.6	4.06±0.5*	4.42±0.4	5.26±0.4
BV/TV	30.67±2.0	14.39±2.6***	20.64±2.9	11.57±3.3
Bone Perimeter (BS) mm	53.82±5.0	20.98±3.6***	32.93±1.1	22.51±5.1
BS/BV (mm^-1^)	30.41±1.4	38.30±2.5*	39.58±2.9	45.22±4.7
BS/TV (mm^-1^)	9.16±0.3	5.20±0.6****	7.77±0.7	4.40±1.0*
Trabecular Thickness (um)	67.46±3.1	54.05±3.7*	52.47±3.4	47.36±4.3
Trabecular Number (mm^-1^)	4.58±0.1	2.60±0.3****	3.89±0.3	2.20±0.5*
Trabecular Space (um)	154.3±8	354.0±49***	218.9±28	674.5±242
Erosion Perimeter (mm)	4.46±0.6	1.55±0.1**	2.76±0.1	1.71±0.4*
Quiescent Perimeter (mm)	49.37±4.5	19.43±3.6***	30.16±1.3	20.80±4.8
Osteoblast Perimeter (mm)	24.22±2.3	8.16±1.9***	14.70±1.2	8.60±2.1*
Osteoclast Perimeter (mm)	4.46±0.6	1.55±0.1**	2.76±0.1	1.71±0.4*
Erosion Perimeter/BS	8.171±0.71	7.932±0.88	8.608±0.70	7.223±0.97
Quiescent Perimeter/BS	91.83±0.7	92.07±0.9	91.34±0.7	92.78±1.0
Osteoblast Perimeter/BS	44.91±0.7	37.69±2.6*	44.14±2.1	36.28±4.7
Osteoclast Perimeter/BS	8.179±0.71	7.932±0.88	8.61±0.70	7.193±0.97
Number of Osteoblasts/BS	34.98±0.5	32.15±2.1	36.74±3.4	31.62±4.6
Number of Osteoclasts/BS	2.383±0.22	2.930±0.37	2.915±0.20	2.649±0.30

(*p <0.05, **p <0.01, ***p <0.001, ****p <0.0001)

**Fig 4:**
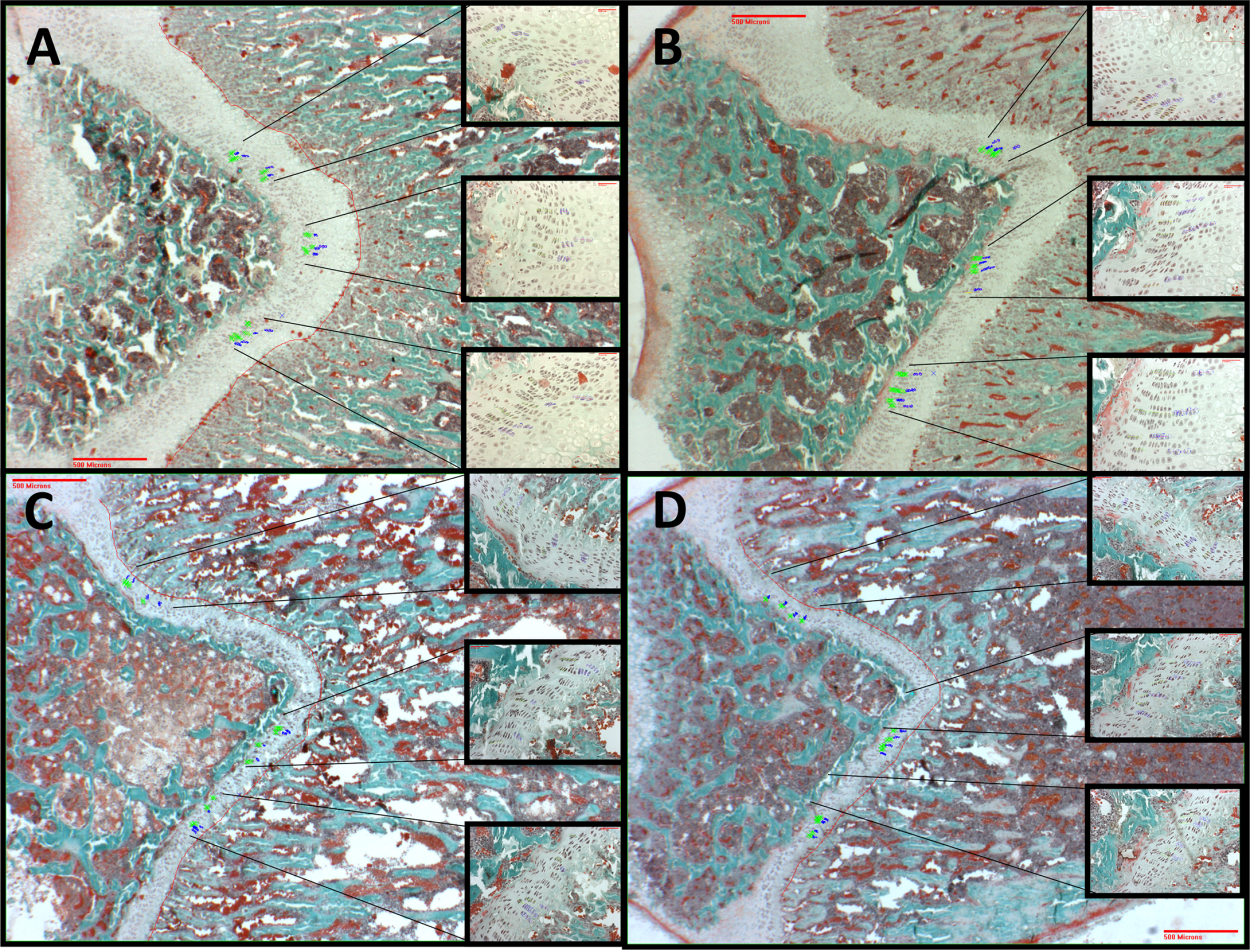
Cartilaginous growth plate analysis. Cartilaginous growth plates were evaluated based on overall growth plate thickness, average proliferating zone thickness, average number of proliferating cells/column, average hypertropic zone thickness, mean volume of hypertrophic cells, and mean hypertrophic cell volume standard deviation. Pictured growth plates are from the rats included in Fig 3 (as well as microCT images in Fig 2). They include 38 day old WT (A) and 42 day old *Cftr-/-* (C) male rats. Female bone images are from 42 day old *Cftr-/-* (B) and WT (D) rats. Each larger image is at 2X, with inserts demonstrating areas of measurement at 20X. Green outlined cells represent the proliferative zone and blue outlined cells are the hypertrophic zone. Both male and female *Cftr-/-* rats demonstrated a reduction in overall growth plate thickness (43% reduction in males and 35% in females). However, in males there was also a reduction in the hypertrophic zone thickness, mean volume of hypertrophic cells and hypertrophic cell volume standard deviation in the *Cftr-/-* rats, but not in the proliferative zone thickness or average number of proliferating cells/column. These findings are suggestive of a difference in the maturation from the proliferative zone into the hypertrophic zone, or differences in cellular activity of the hypertrophic zone, between the *Cftr-/-* and the WT rats.

Histologic comparison of growth plates in male and female rats is demonstrated in Fig 4. Male rats (n = 7 *Cftr-/-* and 6 WT) revealed a 43% reduction in overall femur growth plate thickness of *Cftr-/-* rats (505.2±44.4 vs. 284.4±44.2 μm, p<0.01). We observed no difference in proliferative zone thickness or average number of proliferating cells/column. However, average hypertrophic zone thickness and mean volume of hypertrophic cells were reduced in male *Cftr-/-* rats (39.34±2.35 vs. 30.06±3.00 μm^3^ and 87.53±6.30 vs. 56.58±8.50 μm^3^; p<0.05 for both). In addition, the mean hypertrophic cell volume standard deviation was reduced in *Cftr-/-* rats (39.06±3.58 vs. 27.38±3.73 μm^3^, p<0.05). This suggests difference in maturation from the proliferative zone to the hypertrophic zone, or a reduction in cellular activity in the hypertrophic zone of *Cftr-/-* rats. In female rats (n = 4 *Cftr-/-* and 5 WT), we found a 35% reduction in overall femur growth plate thickness in *Cftr-/-* rats (395.5±16.9 vs. 255.7±49.9 μm, p<0.05). However, differences in the different zones of the female rat growth plate did not achieve significance. The lack of significance is believed to be due to the small size of the growth plates and the number of comparable samples.

### Insulin-like growth factor-I (IGF-I) is reduced in *Cftr-/-* rats

**Fig 5:**
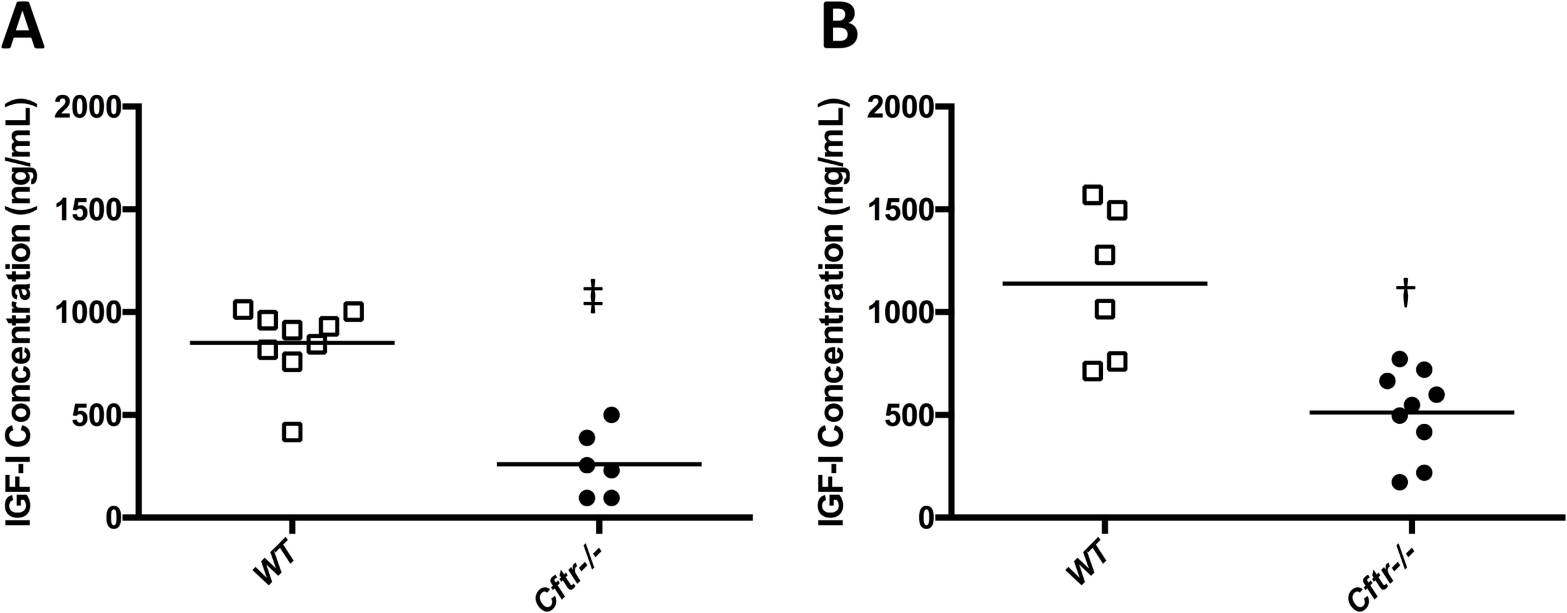
IGF-I is reduced in *Cftr-/-* rats. Serum IGF-I concentrations were reduced in *Cftr-/-* (•) male (261.5 ± 65.7 vs. 850.8 ± 61.1 ng/mL, p<0.0001, (A) and female (512.6 ± 70.1 vs. 1139 ± 149.7 ng/mL, p = 0.001, (B) rats compared to WT (▯). Age and weight adjusted IGF-I concentrations are found in the supplemental figure (S1 Fig).

Based on emerging data suggesting IGF-I deficiency affects CF and can alter cartilage growth, we hypothesized IGF-I may be an underlying cause of bone diminishment in CF. To test this, we measured IGF-I in sera of rats at necropsy. Sera were collected and analyzed from additional rats (both *Cftr-/-* and WT), which were not otherwise evaluated for growth or microCT. Serum concentrations were dramatically reduced in *Cftr-/-* versus WT rats for both male (261.5+65.7 vs. 850.8+61.1 ng/mL, p<0.0001) and female rats (512.6+70.1 vs. 1139+149.7 ng/mL, p = 0.001) (Fig 5). Because differences in IGF-I concentrations can be seen in relation to weight and age of the animals studied, we further compared the age and weights of both WT and *Cftr-/-* rats to their IGF-I concentrations, to determine whether our findings were secondary to weight differences between the two groups. IGF-I concentrations in both male and female WT rats correlated with age (p = 0.009 and 0.03 respectively) and weight (p = 0.0095 and 0.018), but this association was not observed for *Cftr-/-* animals (these correlations of weight and age to IGF-I are found in S1 Fig). In total, these findings demonstrate an important and significant deficiency of IGF-I in CF rats that is correlated to growth.

## Discussion

Not only is growth restriction an important detrimental outcome associated with CF, but it has also been suggested to directly contribute to overall health. Linear growth has implications concerning lung health, and growth restriction has shown to be an independent predictor of mortality [16]. Reduced growth, prior to and during puberty, correlates with lung disease severity [17]. Interestingly, this finding begins prior to lung impairment, as documented by Konstan et al [18]. Furthermore, height, weight and percent ideal body weight at age three are predictors of pulmonary function by age six. Growth also appears to be related to the severity of the CFTR geneotype, suggesting a link to the functionality of the CFTR. The 2015 CFF Annual Data Report implies that reduced growth is CFTR mutation class dependent—subsequently CFTR activity—and is not necessarily related to weight [1]. Most recently, children with the G551D CFTR genotype demonstrated improved height z-scores and weight z-scores, as well as height and weight growth velocities, when treated with ivacaftor, a CFTR potentiator, which strongly suggests a CFTR dependence to these findings [19].

Given the multifactorial nature of the disease, discriminating between these clinical disease contributions is necessary to appreciate an intrinsic defect due to CFTR-deficiency. A major advantage of using CF animal models is the elimination of variables which may complicate a similar analysis in human subjects. During the first 6-weeks of life, the CF rat does not spontaneously develop pancreatic or lung disease [13]. The CF rat phenotype therefore allows for study of growth and bone metabolism without many of the confounding variables (exocrine pancreatic insufficiency, hepatic disease, respiratory failure) that complicate interpretation of bone disease in the clinical scenario. In the present experiments, we demonstrate that in the absence of other exocrine disease, a defect in linear bone growth and bone health in CF animal models is readily apparent. The length and volume of femurs were significantly reduced compared to WT rats. There was also evidence of diminished bone microarchitecture. We observed not only reduced trabecular volume, but also reduced trabecular number and increased spacing between trabeculae (reducing the structural component of bone, which may predispose to bone breakage). Understandably, the size of the bones reflected the bone content. In general, these findings are consistent with prior studies in CF mice, alghough better elucidating mechanisms and define the nature of the histopathologic abnormality.

An intrinsic defect in bone growth, caused by absence of CFTR, is yet to be determined. However, the findings in the CF rat clearly support this possibility. Evidence for direct dependence on bone metabolism due to CFTR activity has been suggested previously by our laboratory [12], and interventions to improve CFTR function in the murine model have resulted in increased bone mass [20]. Recent work by our group demonstrated that murine osteoblasts, responsible for new bone formation, express CFTR [12]. Furthermore, the lack of CFTR resulted in reduced proliferation and differentiation of the osteoblasts *in vitro*. Although this finding could help explain decreased bone formation in CFBD, the observation cannot account in full for growth restriction in CF rats, since linear growth occurs first through cartilaginous proliferation at the growth plate.

Our findings of reduced size of the overall growth plate—and the hypertrophic cells in the growth plate—provide a bridge between bone disease and growth in CF. Previously, we also reported a decrease in canonical Wnt signaling in *Cftr-/-* osteoblasts [12], and note that increased Wnt signaling may improve osteoblast differentiaton and function in F508del-CFTR mice [21]. Furthermore, canonical Wnt signaling is a major contributor to linear bone growth [22]. Interestingly, functional expression of CFTR has been described in murine chondrocytes [23], which like osteoblasts are differentiated from mesenchymal stem cells. Along with these findings, cartilaginous defects have been observed in the trachea CF mice, rats, and pigs [13, 24, 25]. Taken together, our data could suggest that poor growth in CF is due—at least in part—to defects in the cartilaginous growth plate or through growth factors impacting upon it. Previous studies have shown that IGF-I can play an important role in chondrocyte proliferation and hypertrophy in the growth plate [26, 27]. The reduced levels of serum IGF-I observed in the *Cftr-/-* rats may inpart contribute to the reduced volume of cells in the hypertrophic region of the growth plate. The relationship of the GH/IGF-I axis in linear growth and bone health is well established [28], and treatment with human GH (hGH) has demonstrated improved growth in CF [29]. IGF-I treatment has been utilized to improve linear growth in non-CF children [30], and improves bone density in disease states with reduced anabolic drive (such as anorexia) [31]. More recent studies are examining therapeutic ways to improve cartilage growth and repair with IGF-I [32, 33]. Although IGF-I and/or GH supplementation have not been studied in CF animal models (specifically evaluating bone metabolism and cartilage growth), our data suggest the importance of such studies to improve understanding of growth mechanism.

Our study further expands on the multiple studies in CF mice, which have revealed poor growth and reductions in bone density which can be attributed to the loss of a functional CFTR channel [8, 10, 34–36]. This finding is evident in adult CFTR knockout mice [11], as well as in the gut-corrected CF murine model (designed to eliminate the nutritional deficiency in CF animals) [37]. Interestingly, Rogan et al. demonstrated at birth that CF pigs had reduced humeral length and bone mineral content [9]. In this example, differences in growth and bone occur despite comparable nutritional intake *in utero*, suggesting that these events may begin very early in development.

Also similar to our previous findings in *Cftr-/-* mice [37], reductions in bone content appear more prevalent in female CF rats compared to their male counterparts. However, in the clinical setting, CFBD affects men and women equally. Evidence from the CF mouse model implicates a hypothalamic hypogonadotropic state, resulting in reversible decreased fertility (reduced uterine and ovarian size, decreased ovulation rates and abnormal estrous cycles) [38]. Although studies evaluating estrogen and ovarian function in the CF rats have yet to be performed, differences in bone content and histomorphometry could be related to a potential hypogonadotropic state in the CF rats (similar to the mouse model). However, rats develop sexual maturity between 6–8 weeks of age, with an average pubertal onset of 50 days [39]. In attempts to avoid the influence of pubertal factors or hypogonadism following puberty, animals studied in this experiment did not exceed 42 days of age (6 weeks).

In the current report, we did not observe evidence of increased bone loss that has been noted for for human CFBD. While differences were seen in total osteoclast perimeter, there were no differences in the osteoclast perimeter to total bone perimeter ratio seen on histomorphometry. While micro-CT imaging did demonstrate smaller and less total bone, a reduction in the bone density itself was not seen. Since these animals are generally healthy, we speculate that increased bone resorption may be heavily dependent upon other manifestations of the disease (such as chronic inflammation, medications, vitamin D defiency, etc.).

As the life expectancy of CF individuals has increased, chronic complications of the disease have become increasingly prominent. Growth restriction and CFBD are emerging as common manifestations with significant impact on patient outcomes [2]. In summary, the CF rat model provides new and important evidence for an intrinsic defect in linear bone growth and bone mineral accrural, along with evidence to support the lack of anabolic drive. Bone growth may be an important therapeutic target in CF, and represents another previsouly unsuspected manifestation of the CFTR deficiency.

## Supporting information

S1 Fig IGF-I concentrations compared to age and weight of CF and WT rats. IGF-I concentrations in both male and female WT rats (☐) correlated with age (A and B, p = 0.009 and 0.03 respectively) and weight (C and D, p = 0.0095 and 0.018), but this association was not observed for *Cftr-/-* rats (•).
(PDF)
