# Supplementary material for: Reduced bone length, growth plate thickness, bone content, and IGF-I as a model for poor growth in the CFTR-deficient rat
Source: PLoS One. 2017 Nov 30;12(11):e0188497. doi: 10.1371/journal.pone.0188497 (PMC5708703; doi:10.1371/journal.pone.0188497)
Supplement: S1 Fig — IGF-I concentrations in both male and female WT rats (☐) correlated with age (A and B, p = 0.009 and 0.03 respectively) and weight (C and D, p = 0.0095 and 0.018), but this association was not observed for Cftr-/- rats (•). (PDF) [file pone.0188497.s001.pdf]

Supplemental Figure

IGF-I Concentrations Compared to Age and Weight of CF and WT Rats

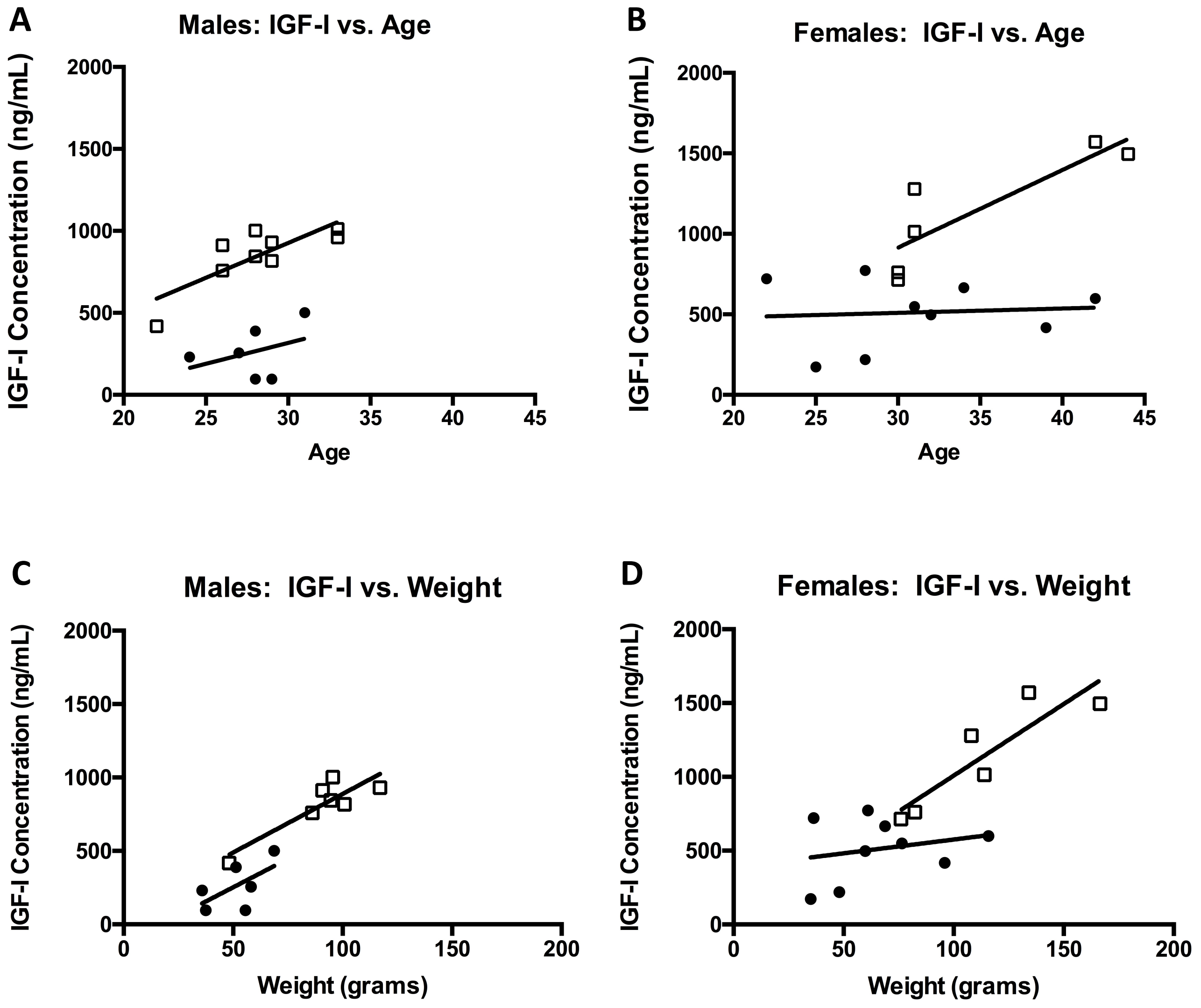

IGF-I concentrations in both male and female WT rats (☐) correlated with age (A and B, p=0.009 and 0.03 respectively) and weight (C and D, p=0.0095 and 0.018), but this association was not observed for *Cftr-/-* rats (●).
